# The impact of an insecticide treated bednet campaign on all-cause child mortality: A geospatial impact evaluation from the Democratic Republic of Congo

**DOI:** 10.1371/journal.pone.0212890

**Published:** 2019-02-22

**Authors:** Carrie B. Dolan, Ariel BenYishay, Karen A. Grépin, Jeffery C. Tanner, April D. Kimmel, David C. Wheeler, Gordon C. McCord

**Affiliations:** 1 Department of Kinesiology and Health Sciences, William and Mary, Williamsburg, Virginia, United States of America; 2 Department of Economics, William and Mary, Williamsburg, Virginia, United States of America; 3 Department of Health Sciences, Wilfrid Laurier University, Waterloo, Ontario, Canada; 4 Independent Evaluation Group, World Bank, Washington, DC, United States of America; 5 Health Behavior and Policy, Virginia Commonwealth University, Richmond, Virginia, United States of America; 6 Department of Biostatistics, Virginia Commonwealth University, Richmond, Virginia, United States of America; 7 School of Global Policy and Strategy, University of California San Diego, San Diego, California, United States of America; Instituto Rene Rachou, BRAZIL

## Abstract

**Objective:**

To test the impact of a nationwide Long-Lasting Insecticidal Nets [LLINs] distribution program in the Democratic Republic of Congo [DRC] on all-cause under-five child mortality exploiting subnational variation in malaria endemicity and the timing in the scale-up of the program across provinces.

**Design:**

Geospatial Impact Evaluation using a difference-in-differences approach.

**Setting:**

Democratic Republic of the Congo.

**Participants:**

52,656 children sampled in the 2007 and 2013/2014 DRC Demographic and Health Surveys.

**Interventions:**

The analysis provides plausibly causal estimates of both average treatment effects of the LLIN distribution campaign and geospatial heterogeneity in these effects based on malaria endemicity. It compares the under-five, all-cause mortality for children pre- and post-LLIN campaign relative to children in those areas that had not yet been exposed to the campaign using a difference-in-differences model and controlling for year- and province-fixed effects, and province-level trends in mortality.

**Results:**

We find that the campaign led to a 41% decline [3.7 percentage points, 95% CI 1.3 to 6.0] in under-5 mortality risk among children living in rural areas with malaria ecology above the sample median. Results were robust to controlling for household assets and the presence of other health aid programs. No effect was detected in children living in areas with malaria ecology below the median.

**Conclusion:**

The findings of this paper make important contributions to the evidence base for the effectiveness of large scale-national LLIN campaigns against malaria. We found that the program was effective in areas of the DRC with the highest underlying risk of malaria. Targeting bednets to areas with greatest underlying risk for malaria may help to increase the efficiency of increasingly limited malaria resources but should be balanced against other malaria control concerns.

## Introduction

Malaria remains a major global health concern, and one where progress has recently stalled[1]. In 2016, approximately 216 million cases of malaria occurred worldwide, an increase of 5 million cases from the previous year[1]. Malaria exacts a disproportionately high burden in Sub-Saharan Africa [SSA], where two countries, Nigeria and the Democratic Republic of the Congo [DRC], account for more than 37% of the global total of estimated malaria cases[1]. Of particular relevance is the DRC, where 40% of deaths among Congolese children are attributed to malaria[2]. The DRC was once renowned in Africa for its clinics, quality of physicians, and primary healthcare systems[3]. Since 1996, however, the DRC has experienced devastating and destabilizing conflict characterized by extreme violence, mass population displacements, and a collapse of public health services[3,4]. Gaining a better understanding of the impact of the existing efforts in the DRC is therefore an important priority for researchers and policymakers alike.

Significant efforts have been made by the international community to reduce the global malaria burden, and many donors are contributing to malaria control interventions, such as with long-lasting insecticide-treated bednets [LLINs], indoor residual spraying [IRS], and artemisinin-combination therapy [ACT][5,6]. Donor support for malaria control programs grew rapidly during the late 2000s[5,6]. The World Health Organization [WHO] estimates that between 2000–2015, the scale-up of such interventions as LLINs, IRS, and anti-malarial drugs like ACT has been associated with a 66% reduction in the malaria death rate in Africa [153 per 100,000 to 52 per 100,000] among all age groups, and a 71% reduction among children under five [7.84 per 100,000 to 2.26 per 100,000] [7]. However, these estimates of changes in malaria mortality are modeled estimates based on intervention coverage and intervention effectiveness derived from clinical trials, which could differ under real world conditions[8,9]. While the evidence of the effectiveness of specific malaria interventions from well-controlled studies is strong,[10] the evidence of the impact of malaria control interventions at the population-level on health outcomes has been mixed.

In cross-country studies, increased development assistance for malaria has been associated with increased rates of bednet ownership and lower estimates of child mortality[11,12]. The implementation of the President’s Malaria Initiative [PMI] has been shown to be associated with lower child mortality rates in Sub-Saharan African countries[13]. Similarly, higher levels of bednet ownership, as measured using national household surveys, is associated with lower levels of parasitaemia and mortality of children between 1–5 years of age across a subset of African countries[14]. However, cross-national studies rely upon comparisons across countries that may differ markedly in terms of malaria burden, including factors that may have shaped the decision of donors to invest in malaria programs in those countries in the first place. Cross-country studies are also likely to be confounded by important omitted variables such as quality of governance, the strength of the health system, or the presence of other disease control programs, all of which could also over or understate the impact of bednet interventions. While other work is similar to ours in using the Demographic and Health Surveys to measure the association between malaria interventions and child mortality,^12^ ours goes beyond evaluating changes at country level [in funding for malaria control, for example] to evaluate staggered scale-up of malaria control efforts in a subnational analysis. The DHS has also been used for subnational analysis at continental scale to model the association between expansion in malaria control measures and reduction malaria incidence,[9] but this work does not measure an effect on child mortality.

Evidence on the impact of malaria control activities and improvements in malaria outcomes at the sub-national level in individual countries also remains mixed. For example, facility-based estimates in Kenya have suggested important declines in malaria mortality throughout the country [15,16]. However, the timing of these declines does not correspond well to the timing of the introduction of malaria control interventions [16]. In addition, facility-level data may be biased if the quality of services delivered or reporting improved over the same time period as the scale-up of malaria control efforts [17]. There is also evidence that malaria may have returned to some areas of Kenya despite continually high rates of malaria interventions [18]. One study suggests that the rapid intensification of malaria control efforts in high-burden areas in Kenya can be associated with declines in malaria, but not everywhere [19].

Outside of Kenya, evidence from other countries is also mixed. The rapid scale-up of ACTs and LLINs in Zanzibar and Equatorial Guinea are associated with very large declines in the incidence of malaria cases [20–22]. Small islands, however, may be special cases with regards to the effectiveness of malaria control efforts. In Botswana, the scale-up of interventions were spatially associated with important declines in malaria cases and mortality [23,24]. Country-level studies in Togo[25] and Malawi[26] that make sound attempts to control for endogeneity suggest bednets induced reductions in mortality for some sub-populations of under-five children. However, many countries have documented no change or even increased malaria burden rates over periods of well-documented scale-up of malaria control programs [27–32]. The scale-up of bednets using a mass campaign in Nigeria have been associated with protection against malaria cases in some districts, but not others [33]. Despite the very high burden of disease from malaria in central African countries, very few studies have investigated the impact of malaria control efforts in this region [17]. Taken together, these studies suggest there is also a need to better understand the variation in both the timing of malaria control scale-up and geographic variation in malaria risk.

Given the mixed evidence, national policymakers need more robust evidence to clarify what services and programs to offer and how to target them–in particular at the subnational level [34]. The increased availability of high-resolution spatial data on the epidemiology of malaria and intervention coverage is also shifting our perspectives concerning how to respond to malaria and how to evaluate these efforts [35–37]. A recent study combined large-sample spatial and temporal data on malaria burden and coverage of malaria control interventions to evaluate interventions in a subset of African countries and thereby illustrating the substantial variation that exists within countries [38]. The authors also found that, at the individual level, the uptake of LLINs and IRS was associated with significant declines in malaria parasitaemia in some regions, but the overall effects were not always significant at the country level. They also found wide ranges of effectiveness for different strategies and suggested that local endemicity might help explain some of these differences. However, the study was also not able to attribute any of the changes of malaria intervention to particular programs or interventions.

In this study, we measure the impact of a nationwide LLIN distribution campaign on all-cause child mortality, focusing on areas where malaria ecology predicts higher malaria burden in the DRC. Unlike previous studies, this research focuses on a specific country but uses variation in the scale-up of malaria control interventions as well as variation in malaria ecology, while also controlling sub-nationally for other aid spending, both province- and year-fixed effects, and other time trends. This approach allows for an explicit and testable hypothesis that geographic location is associated with the impacts on under-five, all-cause child mortality in the DRC[39]. The results complement the existing literature, particularly in informing policy discussions regarding external financing for large-scale deployment of LLINs.

## Methods

The empirical results presented in subsequent sections combine data from four sources: the DRC Demographic Health Survey [DDHS], PMI, the Malaria Ecology Index [MEI], and the AidData Aid Management Platform [AMP]. The authors complied with the conditions of use for each of the four datasets used in the analysis.

### Ethics statement

This research utilizes secondary data which was de-identified and/or aggregated before they were accessed for this study, therefore IRB approval is not required.

### DRC Demographic Health Survey

#### All-cause child mortality

DRC Demographic Health Surveys conducted in 2007 and 2013 serve as our primary source for all-cause child mortality. The DRC has not fielded a Malaria Indicator Survey that would provide data on all internationally recognized malaria indicators. Instead, we rely on a respondent’s complete child birth and death history extracted from a nationally representative sample of women. These birth histories are widely used to measure the mortality of children younger than the age of five in developing countries [S1 Appendix]. The unit of observation is the child level, such that the research design compares differences across children’s mortality risk during the first five years of life. We made use of the geo-referenced data available from the 2007 and 2013/14 DRC Demographic Health Surveys by merging the birth recode files with the GPS data file at the child level. Rajaratnam et al [2010] has extended valuable methodological advancements in model-based approaches to adjust data on summary birth history at a micro-level, however, we did not employ these methods for this project, as they may not be able to measure short-term fluctuations in mortality, a requirement for our identification strategy[40].

The DHS does not document cause of death, so like other studies, we instead focus on all-cause mortality[40]. Given that malaria infection may lead to mortality due to other causes [such as malnutrition if parasitemic subsistence farmers cannot adequately farm their land], this measure captures broader effect of malaria infection beyond mortality directly attributable to the disease.

#### Urban or rural classification

In order to account for consistent inequities in ITN distribution and the fact that malaria is transmitted at lower in rates in urban areas due to a lack of breeding sites for the Anopheles, we included a classification for urban or rural location at the time of interview, as defined by the DHS [41]. In the DHS, urban-rural residence is defined by each country’s national statistical office at the time of each survey. As a robustness check, we confirmed that our results were consistent when using the Global Rural-Urban Mapping Project [GRUMP] urban extent grid [~1km] to define urban and rural location at the time of the interview. The results described below are robust using either the DHS or GRUMP definition of urban areas.

#### Wealth

The asset index provided by the DHS is generally seen as an indicator of long-term wealth and is less volatile than income or consumption. The index is a scale-independent measure of relative socio-economic status that is only valid within a single survey round, since the relative weights of household assets that go into the index change across rounds. Given that we are pooling our data across two survey rounds, we needed a measure of socio-economic status that is consistent across rounds. Therefore, we created an index which has a consistent set of variables across waves, applies the factor loadings from the first wave to the second, and corrects for time-inconsistent response options. The household asset index is the first principal component of a Filmer and Pritchett[42] styled index of 48 comparable household wealth variables contained in both the 2007 and 2013/14 DHS surveys covering water source, type of sanitation, type of floor, type of roof, type of cooking fuel, number of household members per sleeping room, number and relations of adults, and whether or not the household has electricity, radio, a television, a refrigerator, a telephone, a mobile phone, a bicycle, a motorcycle/scooter, or a car/truck. The item weights for the 2007 wave are constant for the 2013–2014 wave to facilitate comparison across time and households [akin to using a price deflator].

In addition to these control variables, we include province-fixed effects that control for unobserved time-invariant province characteristics that may be associated with net rollouts and mortality. Finally, we add province-specific linear trends to control for different mortality trends across provinces prior to the implementation of the LLIN campaign.

### Treatment: Long Lasting Insecticidal Net campaign

**Fig 1:**
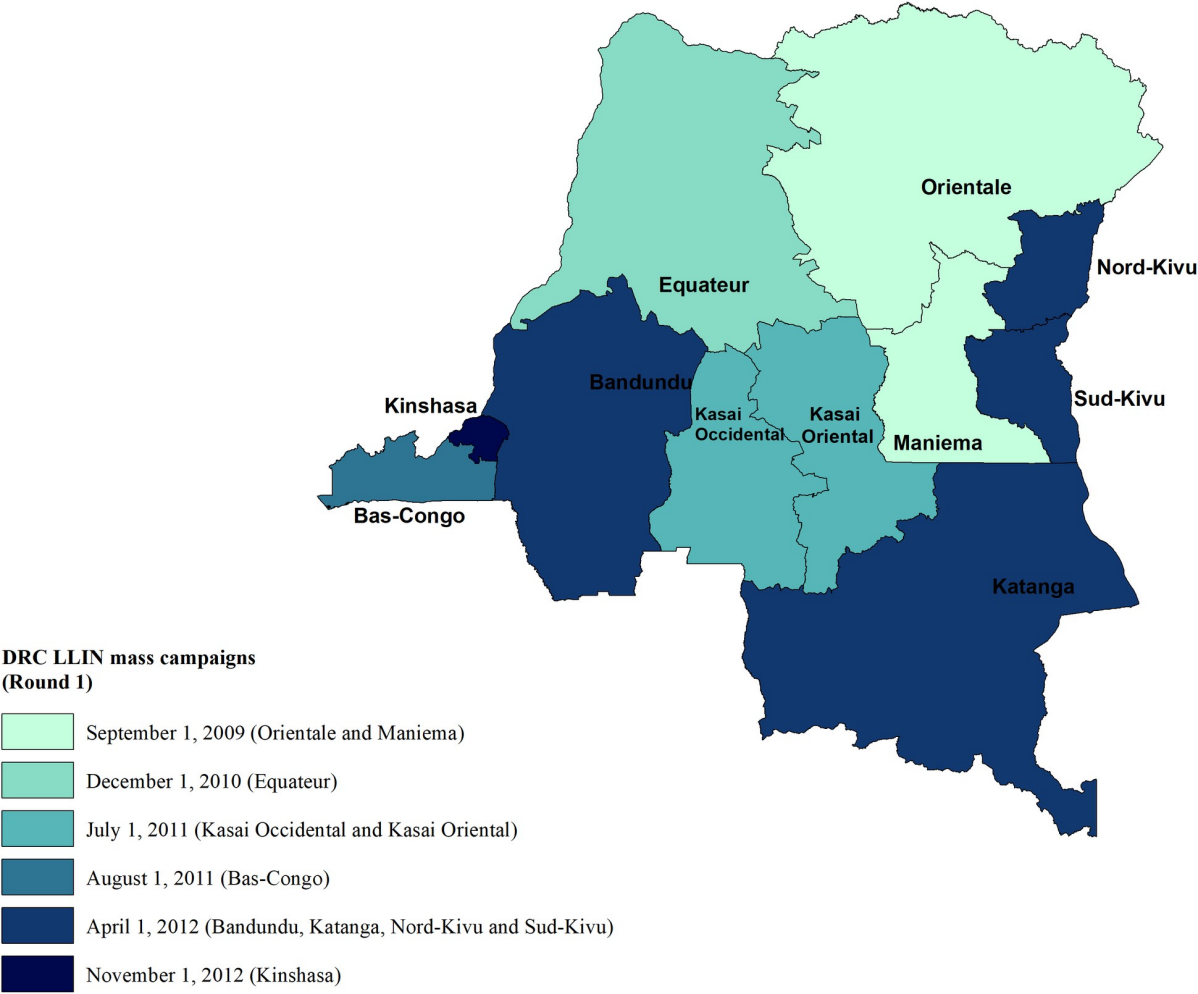
Dates of long-lasting insecticide treated net mass campaigns.

The LLIN campaign is not one campaign, but an ongoing series of mass distributions and replacements at the province level coordinated by the National Malaria Control Program [NMCP]. The treatment is an indicator variable of whether or not the NMCP had reached the province where a child lives during the observed time period. The timing of the LLIN campaigns is available at the month-province level. There are six time periods of campaign rollouts in the eleven DRC provinces [Fig 1]. The treatment variable is based on the timing of the LLIN campaign, not whether a child received a net. All children were considered treated after the date at which campaigns were launched in their province, thus applying the intention to treat principle. We took this approach because we are interested in the population-level effects of the LLIN campaign [which did not achieve 100% coverage], and because even children who do not sleep under nets may still benefit from campaigns through a reduction in mosquitoes[10]. Data from the US government’s PMI program provides information on the timing and location of LLIN funding between 2009 and 2013, as well as the funding partners for each province. The data originates from a centralized database designed to monitor donor coordination and implementation of the DRC National Malaria Strategic Plan through the NMCP.

The LLIN campaign was implemented by the National Malaria Control Program in cooperation and with support from key international donors. While the NMCP also included other prevention strategies, the focus in our study time period was on the distribution of LLINs. From 2009–13, international donors rapidly increased funding to the NMCP, in particular to provide funding to procure and distribute 35 million LLINs covering a population of more than 71 million people[43]. The strategy of this large-scale campaign was to distribute 1–3 LLINs per household, for free, together with instructions for use and information concerning their usefulness [44]. S2 Appendix and S3 Appendix provide a detailed description of the NMCP as well as donor support to the NMCP including amounts given health zones covered, and timing of the onset of the program.

### Malaria Ecology Index

#### Malaria risk

**Fig 2:**
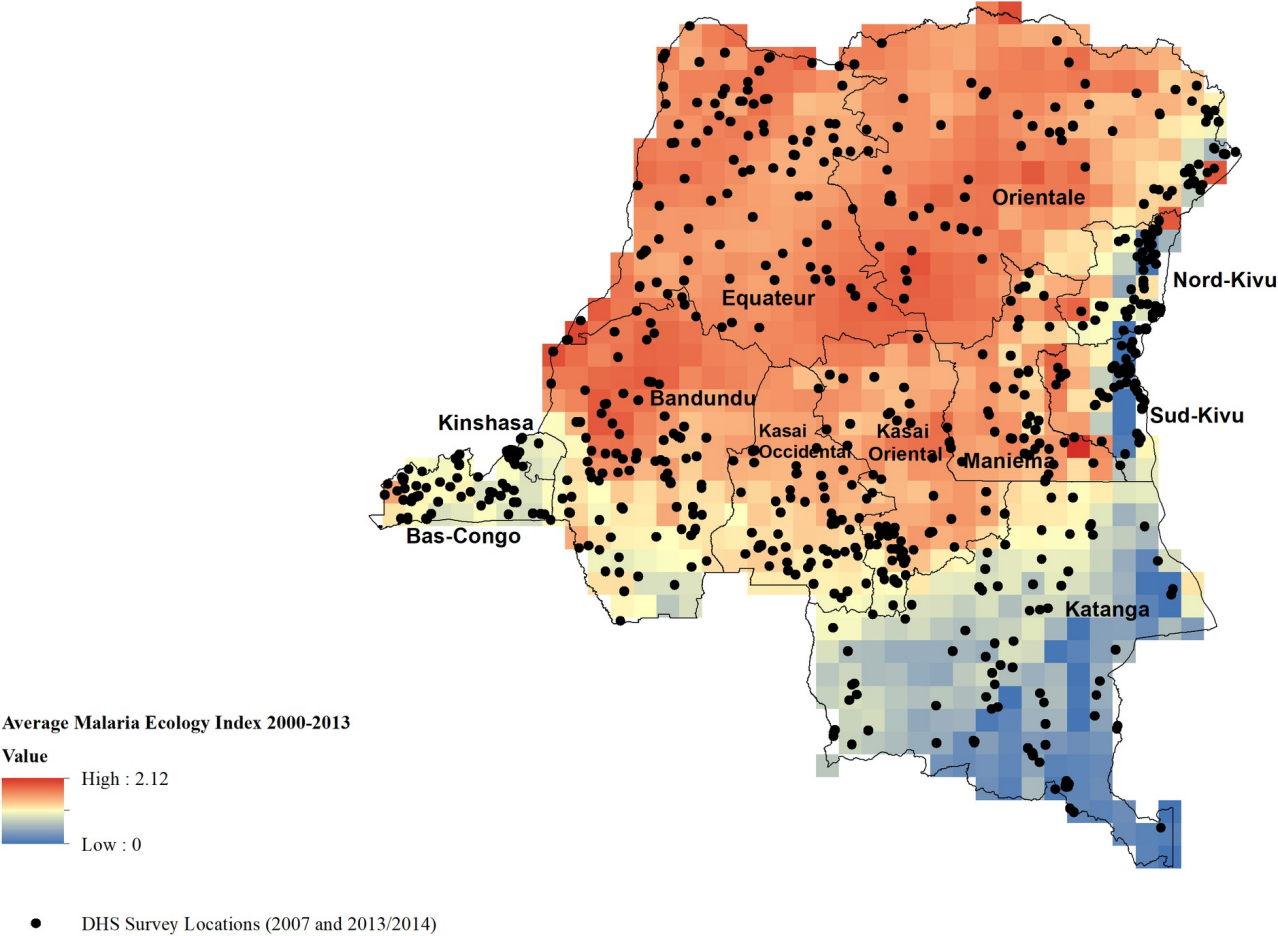
Average Malaria Ecology Index and Demographic Health Survey sampling locations.

Disease ecology, and in turn, the relationship between people and the environment is important for human health [45]. To reflect the underlying malaria risk, one would ideally rely on case data; however, in SSA, weak surveillance systems detect only an estimated 10% of malaria cases [46]. Instead, we use the Malaria Ecology Index [MEI] published elsewhere,[47,48] which takes into account ecological and biological factors affecting the stability of malaria transmission for every month at a spatial resolution of 0.5 decimal degrees [approximately 50km at the equator]. Specifically, the MEI uses the human biting preference of each location’s dominant species of *Anopheles* [the locally dominant vector can vary across months according to precipitation] together with monthly temperature data and temperature’s effect on the extrinsic incubation period of the Plasmodium inside the mosquito’s gut. The MEI measures intensity of transmission for every month, and it has been shown to be strongly associated with malaria outcomes [48]. We merged the geographic location of each household to the gridded MEI for each child-month in our sample. The MEI varies month-to-month according to temperature and precipitation; the average MEI during the study period is mapped in relation to the DHS clusters in Fig 2, showing the differences in average transmission strengths across 0.5 degree cells throughout the country. The range of the monthly MEI is 0–2.65, and on average is much higher in the north and west of the country as compared to the south and east. Note that the MEI was constructed at 50 km resolution, which means that there is significant variation within provinces, but it is at low enough resolution that the DHS random displacement [0–5 km in rural areas] is not a major concern. We also limit the sample to children who experience MEI levels above the median in order to measure campaign effects in areas with high ecological force of transmission. This recognizes variation in mortality decreases associated with living in provinces with an active LLIN campaign across areas with high and low malarial burden [49–51].

### Additional financial support

We extracted a summary measure of additional financial support for health initiatives from the DRC Aid Management Platform [AMP], formulated as a binary indicator for whether health aid project funding was provided. The DRC AMP version 12 contains precise locations for 27 health projects at 348 locations in the DRC and represents 8 funders [DfID, USAID, the Embassy of Japan, the Embassy of Belgium, the Embassy of Canada, the Embassy of Sweden, the European Commission, and the Korea International Cooperation Agency]. Despite the advantage of geographically referenced health aid, there are known limitations to this data. For instance, the AMP does not include details about specific projects. As such, it is not possible to subset the health data to malaria-specific projects. We therefore used these data as a control for local aid investments beyond LLINs that may also impact child mortality and malarial prevention efforts. In this analysis, commitment data on health aid was used as a covariate and was obtained from AidData. A major advantage of AidData over the Organisation for Economic Co-operation and Development’s [OECD] Creditor Reporting System [CRS] is that AidData includes more data from non-DAC bilateral donors, as well as additional multilateral and intergovernmental organizations. In general, commitments have a higher coverage than disbursements, but the data should be interpreted more cautiously than estimates based on disbursements [52].

## Study design

In line with other studies measuring the impact of a public health campaign,[24,38,49–51,53] we utilized a difference-in-difference approach augmented with a geospatial variable of the NMCP LLIN distribution campaign exploiting the different timing and locations of the campaign rollout during 2009–2013. By combining data on the distribution of LLINs and child mortality at precise geographic locations, we control for potential confounding factors correlated across children within a province. Since the program was rolled out differentially across provinces over time, we were able to control for factors that improved child mortality across the entire country over our study period. By integrating data on climatic and ecological drivers of mosquito breeding that vary over time and space, we identified the effects of LLIN distribution in locations where malaria transmission risk is higher. This difference-in-differences approach, augmented with a geospatial variable, compares pre- and post-intervention change in under-five, all-cause child mortality risk using a difference-in-differences model. We include year fixed effects, which flexibly de-trend the mortality data at the country level to prevent spurious correlation with the LLIN campaign rollout, as well as province fixed effects to absorb time-invariant omitted variables that could be correlated both to mortality risk and the timing of LLIN campaign. Finally, we include province-specific linear time trends, which account for differences in mortality trends before the LLIN campaign. Therefore, assuming there is no residual confounding, our estimate provides the causal effect of the LLIN campaign on child mortality risk.

We note that while the DHS gathers information on bednet ownership of households at time of survey, this information is insufficient to assign bednet ownership to children who were born in years between the DHS surveys. However, we can use the two available DHS rounds to confirm that bednet ownership did increase dramatically during the years of the LLIN campaign suggesting the program had been widely implemented as intended across the country [S4 Appendix].

### Empirical specification

Linear probability models were used in a difference-in-differences strategy to estimate the likelihood of all-cause child mortality before 60 months of age using child-level observations. The primary specification was estimated as follows:

$$Mortality_{icpt}=\beta_{1}Treatment_{pt}+\beta_{2}MEI_{ct}+\beta_{3}Female_{i}+\beta_{4}HHHage_{i}+\beta_{5}AllHealthAid_{pt}+\Theta_{t}+\Delta_{p}+\Lambda_{p}t+\epsilon_{icpt}$$

*Mortality_icpt_* is a binary variable denoting the mortality of child *i* living in DHS sample cluster *c* in province *p* in year *t*. *Treatment_pt_* is a binary variable coded as 1 the year when the LLIN campaign occurs in the child’s province and thereafter. *MEI_ct_* is the MEI at the sampling location during that year. The regression controls for the sex of the child and the age of the household head. *AllHealthAid_pt_* is available as a binary variable for each month and year to denote funding from international donors provided to that location. The variable is averaged over the months of the child’s life, and so represents the percentage of months the child’s province benefited from international donor funding. Θ_*t*_ is a time-fixed effect to flexibly absorb national trends in mortality and LLIN coverage that might confound our estimate. Δ_*p*_ is a province-fixed effect that absorbs time-invariant characteristics of provinces that might be correlated with both mortality levels and the timing of LLIN distribution. This province-fixed effect controls for time-invariant cross-province differences in health service provision, and because distribution was assigned to donors at the province level, the model is appropriately identified at the level of variation of implementation. Λ_p_ is a set of province-specific linear trends, which controls for the different trends in mortality that provinces may have before treatment. De-trending the data at the unit of treatment strengthens causal inference in observational studies, given the potential absence of parallel trends in mortality before the campaign in different provinces.

It is important to note that, despite a binary dependent variable, we opt for a linear probability model in our empirical analysis because some of our specifications include interaction terms [for example, interacting with the quintile of assets]. These interactions in combination with location-fixed effects complicates estimation and interpretation using nonlinear models such as proportional hazard or Poisson [54]. The LPM is often used in large-data studies of mortality [24,55]. As a robustness check, we implement a probit version of our main regression specification to show that results are not qualitatively different.

The model can be interpreted as a difference-in-differences estimator of the cross-cohort, cross-province differences in mortality rates based on each province’s exposure to the LLIN campaign. Impacts are identified by comparing differences between older and younger cohorts in the provinces with earlier campaigns against the differences between older and younger cohorts in provinces that have not yet received the campaign. This identification strategy is similar to those of several other recent papers studying mortality reductions [50,53]. All analysis was conducted using Stata, version 14.

## Results

Table 1 presents descriptive statistics on the sample characteristics by treatment status for the primary outcome and control variables for our sample. The child mortality sample from the DHS included 52,656 children, of which 40,167 [76%] were children living in rural areas.

**Table 1:** Summary table by Long Lasting Insecticidal Nets [LLINs] distribution [n = 52,656 children].

Sample characteristics	Children living in provinces with LLIN campaigns^1^ [n = 23,483]		Children living in provinces without LLINs campaigns [n = 29,173]	
	N[%], 2000–2013			
Under five child mortality				
alive	22,823	97.2%	25,646	87.9%
dead	660	2.8%	3,527	12.1%
Malaria Ecology Index	1.46	0.39	1.42	0.43
Rural	18,598	79.2%	21,569	73.9%
urban	4,885	20.8%	7,604	26.1%
Gender of Child				
male	11,585	49.3%	14,738	50.5%
female	11,898	50.7%	14,435	49.5%
Health Aid^2^	0.75	0.73	0.05	0.16
Geographic Classification				
urban	4,830	20.6%	7,278	25.2%
rural	18,598	79.4%	21,569	74.8%
	Mean [SD]			
Asset Index	-2.79	2.6	-1.58	3.07
Age of Household Head*	42.7	10.9	42.6	10.9

^1^ Refers to Long Lasting Insecticidal Nets [LLINs]

^2^ The DRC Aid Management Platform contains precise locations for 27 health projects at 348 locations and represents 8 funders [Department for International Development, USAID, Embassy of Japan, Embassy of Belgium, Embassy of Canada, Embassy of Sweden, European Commission, Korea International Cooperation Agency]

**Fig 3:**
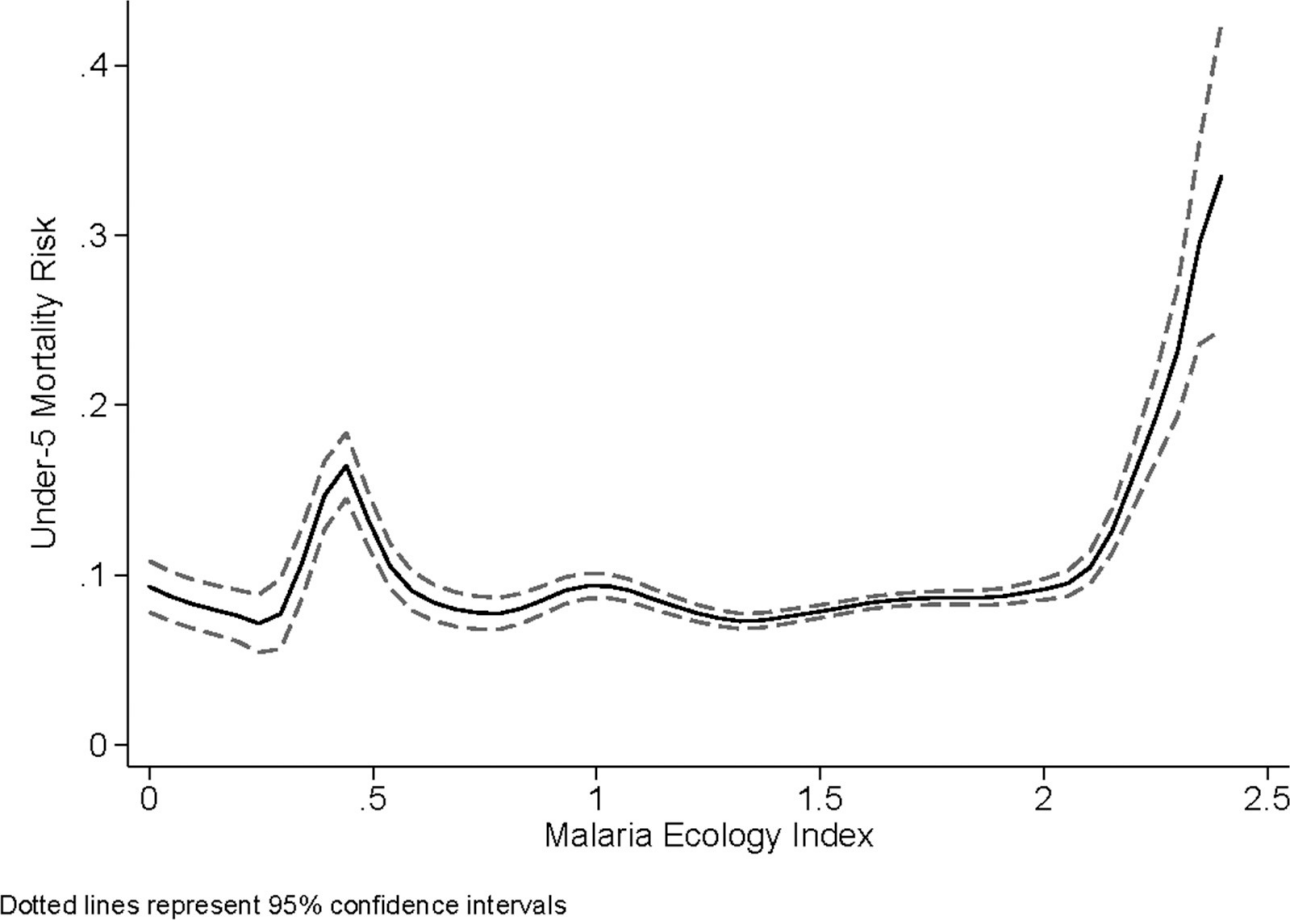
Average mortality risk over the range of average MEI.

Table 2 presents the main results of this analysis. Column [i] presents estimates from a linear probability model for under-five, all-cause mortality for the sample of children living in rural areas [note that the mortality risk in the sample is 8.4%]. This specification finds that there is no statistically significant average treatment effect of the LLIN campaign across the entire sample of children. Fig 3 flexibly plots the average mortality risk over the range of average MEI that children are exposed to in the data and shows that the relationship between all-cause mortality and malaria is only apparent in the upper 50% of MEI values.

**Table 2:** Treatment effect of LLIN campaign on under-5 mortality risk.

	[i]	[ii]	[iii]	[iv]	[v]	[vi]
	LPM	LPM	LPM	LPM	LPM	Probit
Treatment [LLIN^1^ campaign]	0.008	-0.037***	-0.037***	-0.032**	-0.044***	-0.233**
	[-0.028–0.044]	[-0.060 - -0.013]	[-0.059 - -0.015]	[-0.057 - -0.006]	[-0.061 - -0.027]	[-0.455 - -0.010]
Malaria Ecology Index [MEI]	.011					
	[-0.011–0.033]					
Female Child	0.004*	0.006**	0.004	0.006*	0.006*	0.05*
	[-0.001–0.008]	[-0.0003–0.012]	[-0.003–0.011]	[-0.0002–0.012]	[-0.0004–0.012]	[-0.006–0.106]
Age of household head	-0.0001	-0.0002	-0.0002	-0.0002	-0.0002	-0.002
	[-0.0003–0.0002]	[-0.0006–0.0003]	[-0.0006–0.0002]	[-0.0007–0.0002]	[-0.0007–0.0002]	[-0.006–0.001]
Health Aid^2^	0.561***	0.908***	0.907***	0.916***	0.719***	3.836***
	[0.235–0.887]	[0.717–1.10]	[0.731–1.08]	[0.728–1.10]	[0.345–1.09]	[3.19–4.48]
Rural Area			0.013**			
			[0.004–0.023]			
Poorest Quintile in Assets				0.013*		
				[-0.002–0.028]		
Treatment * Poorest Quintile				-0.014*		
				[-0.030–0.002]		
Observations	40,167	20,083	24,849	19,733	20,083	20,083
Sample	Rural	Rural	All	Rural	Rural	Rural
Province FE	YES	YES	YES	YES	YES	YES
Time FE	YES	YES	YES	YES	YES	YES
Province Linear Trends	YES	YES	YES	YES	NO	YES

Dependent variable is mortality of children under 5 years of age

95% confidence intervals in parentheses, clustered to province level to account for spatial autocorrelation in treatment across villages in the same province, as well as serial correlation in the dependent variable in the same province over time

***p<0.01

**p<0.05

*p<0.1

^1^ Long Lasting Insecticidal Nets [LLINs]

^2^ The DRC Aid Management Platform contains precise locations for 27 health projects at 348 locations and represents 8 funders

Column [ii] presents the results of our sub-sample of children which is limited to 50% of children exposed to higher MEI during their first years of life [the cutoff MEI value is 1.55]. The treatment effect is now strongly significant [at p<0.01], and indicates that children living in highly malarious provinces at the time of their birth and that had received the bednet campaign experienced a 3.7 [CI 1.3–6.0] percentage point reduction in mortality [a 41% reduction against an average 9.0% mortality risk in that sample] relative to children living in similar areas that had not yet received the nets. In terms of other variables in the model, the results indicate that female children have a higher mortality risk, that the age of household head is not associated to mortality risk, and that locations where donors engaged in health-related aid project are those with higher mortality [consistent with donors targeting poorer parts of the country].

Column [iii] continues to limit the sample to the upper half of the distribution of MEI experienced by children, but adds children living in urban areas to the sample [and a control for rural location]. The rural location coefficient shows that mortality is higher in those locations, and the effect of the LLIN campaign is unchanged.

**Fig 4:**
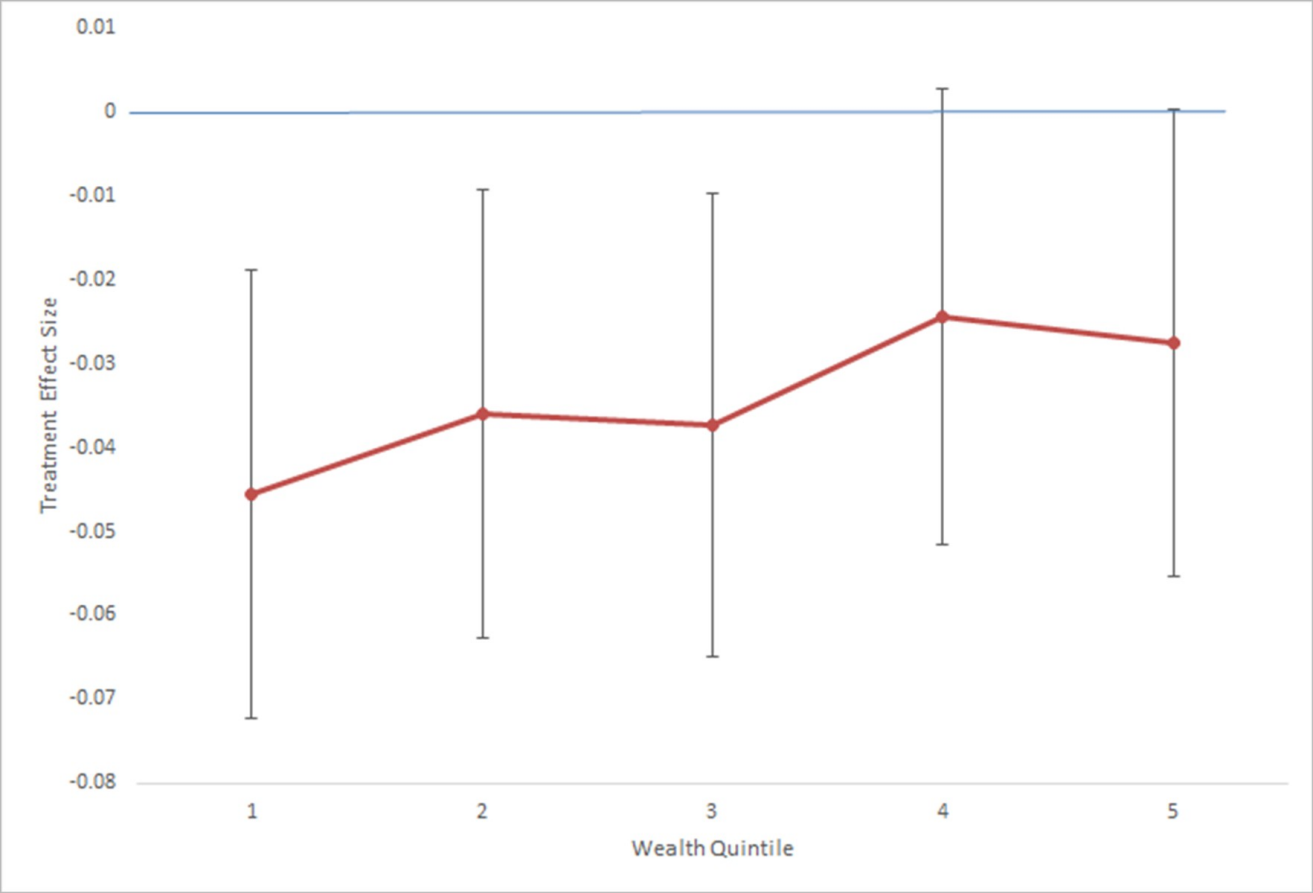
Treatment effect by quintile.

Column [iv] explores whether the poorer parts of the population [in terms of assets measured by the DHS] benefited more or less from the LLIN campaign. The regression adds a binary variable for the lowest asset quintile, and an interaction term of that quintile with the treatment variable. The result suggests that the treatment effect is stronger among poorer households [a 4.6 percentage point mortality risk reduction among the poorest households, compared to 3.2 percentage points among the top four quintiles]. Fig 4 estimates the treatment effect for each quintile, and shows that while the effects are not significantly different from each other, the LLIN campaign reduced mortality at all wealth quintiles in this sample, and the effect shows a pattern of decreasing strength as one moves from poor to rich quintiles.

**Fig 5:**
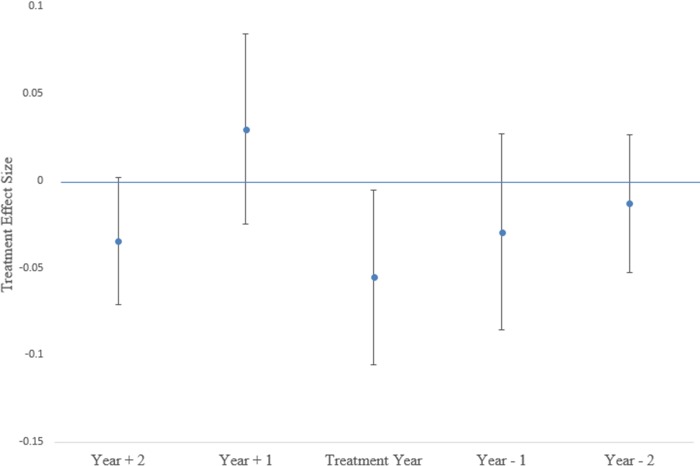
Results of distributed lag-lead model.

The remaining specifications serve as robustness checks. Column [v] removes the province-specific linear trends, and finds that the treatment effect increases in magnitude but is not statistically different from the main result in column [ii]. Column [vi] implements a probit model instead of the linear probability, and shows that the LLIN campaign reduced mortality likelihood. In terms of magnitude, the marginal effect of the treatment variable in the probit model is a 2.6 percentage point decrease [CI 0.1–5.0], and not statistically different from the point estimate in column [ii]. Finally, Fig 5 shows the result of a distributed lag-lead model. The results indicate that the lag effects are insignificant, suggesting that the entire effect of the LLIN campaign on mortality occurred in the same year it arrived at each location. Our empirical approach assumes that any differential gain in survival probability between treatment and comparison provinces that coincides with our treatment variable is attributable to the mass campaign. The lead tests in this Figure provide an indirect test of this assumption by assessing differences in mortality trends during time periods immediately preceding the campaign in each province. If areas that received the mass campaign were already experiencing faster decreases in mortality than areas that received the mass campaign later, this would raise significant concerns about the validity of our results. The leads were not statistically significant, providing further support that improvements in mortality are attributable to the campaign.

## Discussion

This study evaluates the impact of a national LLIN campaign that took place in the DRC from 2009–2013 on all-cause mortality of children under five years of age using nationally representative survey data. We found that the campaign was associated with important declines in child mortality in areas with malaria ecology above the median. Specifically, children living in areas with a bednet campaign and with high measures of malaria transmission experienced a 41% lower rate in all-cause child mortality compared to children in similar areas that had not received the bednet campaign. The magnitude of the decrease is consistent with estimates of around 71% decline in malaria mortality in children under 5 attributed to scale up of malaria control programs.^7^ The results were robust to controls for household assets, the presence of other health aid programs, and limiting the sample to rural areas.

It is important to note that these estimates are intent to treat [ITT] estimates for the total sample. This measures the effect of being made eligible for treatment, since not all households in provinces under the campaign actually end up having nets at home. Moreover, the campaign may take more than one year to reach maximum LLIN coverage in the geographically large provinces of the DRC. This is an important distinction from measuring the treatment on the treated [ToT], that is, the effect of the LLINs on those who received a LLIN. The ITT is the relevant metric to evaluate the effects of mass distribution because the roll out of a program is the lever that is under policy makers’ control instead of the uptake of the intervention at an individual level. The fact that these estimates are ITT partly explain the difference in results compared to experimental studies or those where households with LLINs are compared to those without.

The findings of this paper make some important contributions to the evidence base of the effectiveness of large-scale national LLIN campaigns against malaria. First, it provides evidence that these campaigns can be effective, but in the case of the DRC, significant mortality reduction was evident only in areas where malaria ecology is above the median. The cutoff used in our study [an MEI value of 1.55 and above] represents a malaria ecology prevalent across 51.3% of sub-Saharan Africa’s surface area, speaking to the generalizability of these results. This finding suggests that if donors wish to allocate increasingly scarce resources for malaria to maximize mortality reductions, they should consider prioritizing deployment and continuous coverage of bednets to areas with highest risk of malaria. That said, we recognize there are other benefits of providing malaria control initiatives even in areas at lower levels of endemicity and that setting the threshold arbitrarily at the median MEI could have dire consequences. In particular, it is important to consider the implications for the duration of the campaign as highlighted by Girond et al. [2018] [56]. After about a year the effectiveness of mass distribution campaigns of LLINs seems to decrease while continuous distribution of LLINs maintains a reduction in malaria case notification. This knowledge is essential when considering the pursuit of malaria elimination goals in high transmission areas. Finally, this work also helps to reconcile some of the mixed results from previous studies that did not distinguish between the subnational burdens of malaria.

While our analysis used subnational data on the timing of LLIN distribution, it is important to consider that our estimates could be confounded if the NMCP scaled up LLIN distribution and other malaria interventions to the same provinces in the same years. We cannot rule out the impact of other programs that were implemented in the same provinces at the same time. To explore this further, we used indicators from the two available DHS rounds and found that areas with large increases in bednet usage did not also see increases in usage of ACT or in mothers seeking advice or treatment for feverish children [results in S5 Appendix]. These results suggest that other malaria interventions were not rolled out in the same geographic and temporal manner as the LLIN campaign, which provides evidence that the observed declines in all-cause mortality were mostly likely driven by the rollout of LLINs. Nevertheless, we interpret our estimates of the effect of the LLIN distribution on mortality as representing the effects of the overall public health program, including any activities [staff training or other investments] that went hand-in-hand with the net distribution.

Ending the malaria epidemic is included as one of the Sustainable Development Goals. Despite tremendous progress since 2000, donors and national governments face the formidable challenge of continuing to reduce the disease burden in settings where implementation is difficult and in spite of a decline in growth of funding for malaria and an increase in pyrethroid resistance of the *Anopheles* vector [57]. The most recent data shows that the momentum towards reduction of malaria burden has stalled [1]. Given the urgency of further progress against malaria and the plateauing of donor resources for the disease, targeting of large-scale bednet campaigns in order to prioritize strategically along the lines suggested by this study is becoming increasingly important.

## Supporting information

S1 Appendix Data. (DOCX)

S2 Appendix Detailed description of the NMCP. (DOCX)

S3 Appendix Detailed description of major donor support to the NMCP including amounts given, health zones covered, and timing of the onset of the program. (DOCX)

S4 Appendix Percentage of children under 5 who slept under an ITN the previous night. (DOCX)

S5 Appendix Analysis. (DOCX)

## References

[1] World Health Organization. World Malaria Report 2017. 2017.

[2] International MF. Democratic Republic of the Congo Poverty Reduction Strategy Paper. 2013;13/226.

[3] USAID. Global Health: Democratic Republic of the Congo. 2017; Available at: https://www.usaid.gov/democratic-republic-congo/global-health

[4] Coghlan B, Brennan RJ, Ngoy P, Dofara D, Otto B, Clements M, et al Mortality in the Democratic Republic of Congo: a nationwide survey. The Lancet 2006;367[9504]:44–51. 10.1016/S0140-6736(06)67923-3 16399152

[5] Zelman B, Kiszewski A, Cotter C, Liu J. Costs of eliminating malaria and the impact of the global fund in 34 countries. PLoS One 2014;9[12]:e115714 10.1371/journal.pone.0115714 25551454 PMC4281070

[6] Pigott DM, Atun R, Moyes CL, Hay SI, Gething PW. Funding for malaria control 2006–2010: a comprehensive global assessment. Malaria journal 2012;11[1]:246. 22839432 10.1186/1475-2875-11-246 PMC3444429

[7] World Health Organization. World Malaria Report 2015. 2015.

[8] Gething PW, Battle KE, Bhatt S, Smith DL, Eisele TP, Cibulskis RE, et al Declining malaria in Africa: improving the measurement of progress. Malaria journal 2014;13[1]:39. 24479555 10.1186/1475-2875-13-39 PMC3930350

[9] Bhatt S, Weiss DJ, Cameron E, Bisanzio D, Mappin B, Dalrymple U, et al The effect of malaria control on Plasmodium falciparum in Africa between 2000 and 2015. Nature 2015;526[7572]:207 10.1038/nature15535 26375008 PMC4820050

[10] Lengeler C. Insecticide‐treated bed nets and curtains for preventing malaria. The Cochrane Library 2004. 10.1002/14651858.CD000363.pub2 15106149

[11] Akachi Y, Atun R. Effect of investment in malaria control on child mortality in sub-Saharan Africa in 2002–2008. PLoS One 2011;6[6]:e21309 10.1371/journal.pone.0021309 21738633 PMC3127861

[12] Flaxman AD, Fullman N, Otten MW Jr, Menon M, Cibulskis RE, Ng M, et al Rapid scaling up of insecticide-treated bed net coverage in Africa and its relationship with development assistance for health: a systematic synthesis of supply, distribution, and household survey data. PLoS Medicine 2010;7[8]:e1000328 10.1371/journal.pmed.1000328 20808957 PMC2923089

[13] Jakubowski A, Stearns SC, Kruk ME, Angeles G, Thirumurthy H. The US President’s Malaria Initiative and under-5 child mortality in sub-Saharan Africa: A difference-in-differences analysis. PLoS medicine 2017;14[6]:e1002319 10.1371/journal.pmed.1002319 28609442 PMC5469567

[14] Lim SS, Fullman N, Stokes A, Ravishankar N, Masiye F, Murray CJL, et al Net benefits: a multicountry analysis of observational data examining associations between insecticide-treated mosquito nets and health outcomes. PLoS medicine 2011;8[9]:e1001091 10.1371/journal.pmed.1001091 21909249 PMC3167799

[15] Okiro EA, Alegana VA, Noor AM, Mutheu JJ, Juma E, Snow RW. Malaria pediatric hospitalization between 1999 and 2008 across Kenya. BMC medicine 2009;7[1]:75. 20003178 10.1186/1741-7015-7-75 PMC2802588

[16] Snow RW, Marsh K. Malaria in Africa: progress and prospects in the decade since the Abuja Declaration. Lancet 2010 7 10;376[9735]:137–139. 10.1016/S0140-6736(10)60577-6 20417552 PMC2907486

[17] O'Meara WP, Mangeni JN, Steketee R, Greenwood B. Changes in the burden of malaria in sub-Saharan Africa. The Lancet infectious diseases 2010;10[8]:545–555. 10.1016/S1473-3099(10)70096-7 20637696

[18] Zhou G, Afrane YA, Vardo-Zalik A, Atieli H, Zhong D, Wamae P, et al Changing patterns of malaria epidemiology between 2002 and 2010 in Western Kenya: the fall and rise of malaria. PloS One 2011;6[5]:e20318 10.1371/journal.pone.0020318 21629783 PMC3100336

[19] Pathania V. The impact of malaria control on infant mortality in Kenya. Economic Development and Cultural Change 2014;62[3]:459–487.

[20] Bhattarai A, Ali AS, Kachur SP, Mårtensson A, Abbas AK, Khatib R, et al Impact of artemisinin-based combination therapy and insecticide-treated nets on malaria burden in Zanzibar. PLoS Medicine 2007;4[11]:e309 10.1371/journal.pmed.0040309 17988171 PMC2062481

[21] Aregawi MW, Ali AS, Al-Mafazy A, Molteni F, Katikiti S, Warsame M, et al Reductions in malaria and anaemia case and death burden at hospitals following scale-up of malaria control in Zanzibar, 1999–2008. Malaria journal 2011;10[1]:46. 21332989 10.1186/1475-2875-10-46 PMC3050777

[22] Kleinschmidt I, Schwabe C, Benavente L, Torrez M, Ridl FC, Segura JL, et al Marked increase in child survival after four years of intensive malaria control. Am J Trop Med Hyg 2009;80[6]:882–888. 19478243 PMC3748782

[23] Simon C, Moakofhi K, Mosweunyane T, Jibril HB, Nkomo B, Motlaleng M, et al Malaria control in Botswana, 2008–2012: the path towards elimination. Malaria journal 2013;12[1]:458. 24359262 10.1186/1475-2875-12-458 PMC3893547

[24] Demombynes G, Trommlerová SK. What has driven the decline of infant mortality in Kenya in the 2000s? Economics & Human Biology 2016;21:17–32. 26707059 10.1016/j.ehb.2015.11.004

[25] Ishida K, Stupp P, Erskine M, Goldberg H, Morgah K. The problems of eligibility and endogenous confounders when assessing the mortality impact of a nationwide disease-prevention programme: The case of insecticide-treated nets in Togo. Population studies 2011;65[1]:57–71. 10.1080/00324728.2010.544323 21294055

[26] Deuchert E, Wunsch C. Evaluating nationwide health interventions when standard before-after doesn't work: Malawi's ITN distribution program. 2010.

[27] Coulibaly D, Travassos MA, Kone AK, Tolo Y, Laurens MB, Traore K, et al Stable malaria incidence despite scaling up control strategies in a malaria vaccine-testing site in Mali. Malaria journal 2014;13[1]:374. 25238721 10.1186/1475-2875-13-374 PMC4180968

[28] Mukonka VM, Chanda E, Haque U, Kamuliwo M, Mushinge G, Chileshe J, et al High burden of malaria following scale-up of control interventions in Nchelenge District, Luapula Province, Zambia. Malaria journal 2014;13[1]:153. 24755108 10.1186/1475-2875-13-153 PMC4016669

[29] Okiro EA, Kazembe LN, Kabaria CW, Ligomeka J, Noor AM, Ali D, et al Childhood malaria admission rates to four hospitals in Malawi between 2000 and 2010. PloS one 2013;8[4]:e62214 10.1371/journal.pone.0062214 23638008 PMC3637378

[30] Jagannathan P, Muhindo MK, Kakuru A, Arinaitwe E, Greenhouse B, Tappero J, et al Increasing incidence of malaria in children despite insecticide-treated bed nets and prompt anti-malarial therapy in Tororo, Uganda. Malaria journal 2012;11[1]:435. 23273022 10.1186/1475-2875-11-435 PMC3551700

[31] Landoh ED, Tchamdja P, Saka B, Tint KS, Gitta SN, Wasswa P, et al Morbidity and mortality due to malaria in Est Mono district, Togo, from 2005 to 2010: a times series analysis. Malaria journal 2012;11[1]:389. 23173765 10.1186/1475-2875-11-389 PMC3519571

[32] Louis VR, Schoeps A, Tiendrebéogo J, Beiersmann C, Yé M, Damiba MR, et al An insecticide-treated bed-net campaign and childhood malaria in Burkina Faso. Bull World Health Organ 2015;93[11]:750–758. 10.2471/BLT.14.147702 26549902 PMC4622154

[33] Kyu HH, Georgiades K, Shannon HS, Boyle MH. Evaluation of the association between long-lasting insecticidal nets mass distribution campaigns and child malaria in Nigeria. Malaria journal 2013;12[1]:14. 23297758 10.1186/1475-2875-12-14 PMC3545742

[34] Lewin S, Oxman AD, Lavis JN, Fretheim A. SUPPORT Tools for evidence-informed health Policymaking [STP] 8: Deciding how much confidence to place in a systematic review. Health Research Policy and Systems 2009;7[1]:S8. 20018115 10.1186/1478-4505-7-S1-S8 PMC3271835

[35] Noor AM, Mutheu JJ, Tatem AJ, Hay SI, Snow RW. Insecticide-treated net coverage in Africa: mapping progress in 2000–07. The Lancet 2009;373[9657]:58–67. 10.1016/S0140-6736(08)61596-2 PMC2652031 19019422

[36] Cotter C, Sturrock HJW, Hsiang MS, Liu J, Phillips AA, Hwang J, et al The changing epidemiology of malaria elimination: new strategies for new challenges. The Lancet 2013;382[9895]:900–911. 10.1016/S0140-6736(13)60310-4 PMC10583787 23594387

[37] Noor AM, Kinyoki DK, Mundia CW, Kabaria CW, Mutua JW, Alegana VA, et al The changing risk of Plasmodium falciparum malaria infection in Africa: 2000–10: a spatial and temporal analysis of transmission intensity. The Lancet 2014;383[9930]:1739–1747. 10.1016/S0140-6736(13)62566-0 PMC4030588 24559537

[38] Giardina F, Kasasa S, Sié A, Utzinger J, Tanner M, Vounatsou P. Effects of vector-control interventions on changes in risk of malaria parasitaemia in sub-Saharan Africa: a spatial and temporal analysis. The Lancet Global Health 2014;2[10]:e615. 10.1016/S2214-109X(14)70300-6 25304636

[39] Macintyre S, Ellaway A, Cummins S. Place effects on health: how can we conceptualise, operationalise and measure them? Soc Sci Med 2002;55[1]:125–139. 12137182 10.1016/s0277-9536(01)00214-3

[40] Rajaratnam JK, Tran LN, Lopez AD, Murray CJL. Measuring under-five mortality: validation of new low-cost methods. PLoS Medicine 2010;7[4]:e1000253 10.1371/journal.pmed.1000253 20405055 PMC2854123

[41] Njau JD, Stephenson R, Menon M, Kachur SP, McFarland DA. Exploring the impact of targeted distribution of free bed nets on households bed net ownership, socio-economic disparities and childhood malaria infection rates: analysis of national malaria survey data from three sub-Saharan Africa countries. Malaria journal 2013;12[1]:245. 23855893 10.1186/1475-2875-12-245 PMC3720242

[42] Filmer D, Pritchett LH. Estimating wealth effects without expenditure data—or tears: an application to educational enrollments in states of India. Demography 2001;38[1]:115–132. 11227840 10.1353/dem.2001.0003

[43] President's MI. Malaria Operational Plan FY2014. 2014.

[44] The WB. Project Appraisal Document on a Proposed Grant in the Amount of SDR 99.3 million [US$150 million equivalent] to the Democratic Republic of Congo for a Health Sector Rehabilitation Support Project. 2005.

[45] Mayer JD. Geography, ecology and emerging infectious diseases. Soc Sci Med 2000;50[7]:937–952. 10714918 10.1016/s0277-9536(99)00346-9

[46] World Health Organization. World Malaria Report 2012. 2012.

[47] Kiszewski A, Mellinger A, Spielman A, Malaney P, Sachs SE, Sachs J. A global index representing the stability of malaria transmission. Am J Trop Med Hyg 2004;70[5]:486–498. 15155980

[48] McCord GC, Anttila-Hughes J. A Malaria Ecology Index Predicted Spatial and Temporal Variation of Malaria Burden and Efficacy of Antimalarial Interventions Based on African Serological Data. Am J Trop Med Hyg 2017;96[3]:616–623. 10.4269/ajtmh.16-0602 28070009 PMC5361535

[49] Bleakley H. Malaria eradication in the Americas: A retrospective analysis of childhood exposure. American Economic Journal: Applied Economics 2010;2[2]:1–45. 10.1257/app.2.2.1 PMC3810960 24179596

[50] Cutler D, Fung W, Kremer M, Singhal M, Vogl T. Early-life malaria exposure and adult outcomes: Evidence from malaria eradication in India. American Economic Journal: Applied Economics 2010;2[2]:72–94.

[51] Lucas AM. Malaria eradication and educational attainment: evidence from Paraguay and Sri Lanka. American Economic Journal: Applied Economics 2010;2[2]:46–71. 10.1257/app.2.2.46 23946866 PMC3740749

[52] Grepin KA. HIV donor funding has both boosted and curbed the delivery of different non-HIV health services in sub-Saharan Africa. Health Aff [Millwood] 2012 7;31[7]:1406–1414. 10.1377/hlthaff.2012.0279 22778329

[53] BenYishay A, Kranker K. All-Cause Mortality Reductions from Measles Catchup Campaigns in Africa. J Hum Resour 2015;50[2]:516–547.

[54] Ai C, Norton EC. Interaction terms in logit and probit models. Economics letters 2003;80[1]:123–129.

[55] Pongou R. Why is infant mortality higher in boys than in girls? A new hypothesis based on preconception environment and evidence from a large sample of twins. Demography 2013;50[2]:421–444. 10.1007/s13524-012-0161-5 23151996

[56] Girond F, Madec Y, Kesteman T, Randrianarivelojosia M, Randremanana R, Randriamampionona L, et al Evaluating effectiveness of mass and continuous long-lasting insecticidal net distributions over time in Madagascar: a sentinel surveillance based epidemiological study. EClinicalMedicine 2018;1:62–69. 10.1016/j.eclinm.2018.07.003 30294720 PMC6169794

[57] N'Guessan R, Corbel V, Akogbeto M, Rowland M. Reduced efficacy of insecticide-treated nets and indoor residual spraying for malaria control in pyrethroid resistance area, Benin. Emerg Infect Dis 2007 2;13[2]:199–206. 10.3201/eid1302.060631 17479880 PMC2725864

