# Supplementary material for: The impact of an insecticide treated bednet campaign on all-cause child mortality: A geospatial impact evaluation from the Democratic Republic of Congo
Source: PLoS One. 2019 Feb 22;14(2):e0212890. doi: 10.1371/journal.pone.0212890 (PMC6386397; doi:10.1371/journal.pone.0212890)
Supplement: S1 Appendix — (DOCX) [file pone.0212890.s001.docx]

**S1 Appendix: Data**

**Demographic and Health Surveys**

In addition to collecting nationally representative data on population, health, HIV, and nutrition, each questionnaire records each woman of reproductive age’s complete fertility history. However, the DHS does not provide estimates of disaggregated causes of death. We therefore only investigate all-cause child mortality. An advantage of the DHS is the inclusion of the latitude and longitude at the center of the interview cluster, allowing evaluators to merge geographic data to the surveys. For this research, we merged data from the 2007 and 2013/14 DHS birth recode files with the GPS data file. This allowed us to assign a spatial component to the birth history of all women interviewed in 2007 and 2013/14.

In retrospective surveys, heaping at reported age of deaths is common.^1^ It is important to note that age at death is disproportionately likely to be reported at 0, 12, 24, 36, 48, and 60 months due to rounding and recall error. Although interviewers ask about the age at death in months of each child who has died, respondents may round this age to 12-month intervals. For example, some deaths reported at 12 months may actually have occurred at 10, 11, 13, or 14 months. Bias can be introduced to the extent that age at death results in the net transfer of deaths from one age group to another one. If heaping is due to rounding up, deaths occurring at 10 or 11 months were misreported as 12 months, then results will be biased downward and mortality will be overestimated. The amount of heaping at 12 months may be measured by dividing the number of deaths at 12 months by the average number of deaths at 10, 11, 12, 13, and 14 months. Under the assumption that the actual number of deaths changes linearly from 10 through 14 months, a value greater than 1 indicates heaping at 12 months. In this study, the heaping index at 12 months was 3.4% compared to the median index of heaping in the DHS-II surveys of 5.^1^ There is no way to redistribute individual deaths accurately by changing the age at death. Age heaping is unlikely to be correlated with the campaign timing, but it does introduce measurement error, which could attenuate the coefficients toward zero.

^1^ Curtis SL. Assessment of the Quality of Data Used for Direct Estimation of Infant and Child Mortality in DHS-II Surveys. dhsprogram.com. 1995; published online March. http://www.dhsprogram.com/pubs/pdf/OP3/OP3.pdf (accessed Dec 15, 2016).
