# Supplementary material for: The impact of an insecticide treated bednet campaign on all-cause child mortality: A geospatial impact evaluation from the Democratic Republic of Congo
Source: PLoS One. 2019 Feb 22;14(2):e0212890. doi: 10.1371/journal.pone.0212890 (PMC6386397; doi:10.1371/journal.pone.0212890)
Supplement: S2 Appendix — (DOCX) [file pone.0212890.s002.docx]

**S2 Appendix:** **Detailed description of the NMCP**

The National Malaria Control Programme (NMCP) is a government program of the Democratic Republic of Congo under the guidance of the DRC Ministry of Public Health. NMCP was launched in 2002 following the Abuja declaration to “halve the malaria mortality for Africa’s people by 2010.”^1^ The focus of the NMCP is to strengthen individual level prevention activities, prevention of malaria during pregnancy, improve case management and treatment, and reinforce epidemic management.^1^ Between 2009 and 2013, the primary activity of the NMCP was the free distribution of nearly 35M LLITNs between, covering an estimated population of more than 71M. The design of the project indicated that 1-3 nets per household were to be handed out for free, together with instruction for use and information on their usefulness. The program was executed under the guidance of the DRC Ministry of Public Health that coordinated the efforts of several donors, assigning a donor or donors to be responsible for project implementation in a particular province.

^1^ Roll Back Malaria. The Abuja Declaration and the plan of action. An extract from the African Summit on Roll Back Malaria. www.who.int. 2003. http://www.who.int/malaria/publications/atoz/whocdsrbm200346/en/ (accessed Sept 20, 2016).
