# Supplementary material for: The impact of an insecticide treated bednet campaign on all-cause child mortality: A geospatial impact evaluation from the Democratic Republic of Congo
Source: PLoS One. 2019 Feb 22;14(2):e0212890. doi: 10.1371/journal.pone.0212890 (PMC6386397; doi:10.1371/journal.pone.0212890)
Supplement: S3 Appendix — (DOCX) [file pone.0212890.s003.docx]

**S3 Appendix: Detailed description of major donor support to the NMCP including amounts given, health zones covered, and timing of the onset of the program**

During the study period eight external funders contributed to the DRC NMCP campaigns: the US government’s President’s Malaria Initiative (PMI), UNITAID, United Nations Children's Fund (UNICEF), United States Agency for International Development (USAID), the World Bank, the Global Fund, Population Services International (PSI), and the Department for International Development (DfID).

The below mentioned provided major donor support for malaria control. In 2012, the Global Fund approved a five-year grant (2012-2016) for $212 million for malaria and prevention control activities. Subsequent funding was awarded for 2013 and 2014 in the amount of $85 million of which $55 million was spent on ITNs through mass campaign distribution. The World Bank project on Health Sector Rehabilitation Support began supporting malaria control in 2006 with a four year, $150 million project including $36 million for malaria prevention and treatment in 83 health zones. World Bank provided a considerable amount of additional financing ($113 million) to provide bednets and other support as part of the NMCP initiative in 63 health zones ending in 2013. The Department for International Development awarded approximately $275 million for an integrated health projects in 56 health zones, including a malaria component. $60 million in specific funding for malaria supported ongoing ITN mass campaigns in two to three provinces. The FY2014 budget for PMI was approximately $34 million and focused on implementation activities in 138 health zones within 5 provinces. Data from PMI provides information on the timing and location of LLITN funding between 2009 and 2013, as outlined in Figure 3. The data originate from a centralized database designed to monitor donor coordination and implementation of the DRC National Malaria Strategic Plan through the DRC NMCP.^1^

^1^ President's Malaria Initiative. Malaria Operational Plan FY2014. pmi.gov. https://www.pmi.gov/docs/default-source/default-document-library/malaria-operational-plans/fy14/drc_mop_fy14.pdf?sfvrsn=16 (accessed Sept 20, 2016).
