# Supplementary material for: The impact of an insecticide treated bednet campaign on all-cause child mortality: A geospatial impact evaluation from the Democratic Republic of Congo
Source: PLoS One. 2019 Feb 22;14(2):e0212890. doi: 10.1371/journal.pone.0212890 (PMC6386397; doi:10.1371/journal.pone.0212890)
Supplement: S4 Appendix — (DOCX) [file pone.0212890.s004.docx]

**S4 Appendix: Percentage of children under 5 who slept under an ITN the previous night**

| **DHS Region** | **ITN 2013** | **ITN 2007** | **Difference between 2007 and 2013** | **ITN campaign roll out date** |
| --- | --- | --- | --- | --- |
| Bandundu | 78.7 | 6.1 | 72.6 | 4/1/2012 |
| Bas-Congo | 67.6 | 30.6 | 37 | 8/1/2011 |
| Equateur | 64.5 | 2.4 | 62.1 | 12/1/2010 |
| Kasai-Occidental | 35.9 | 3.8 | 32.1 | 7/1/2011 |
| Kasai-Oriental | 49.8 | 3 | 46.8 | 7/1/2011 |
| Katanga | 60.6 | 6.1 | 54.5 | 4/1/2012 |
| Kinshasa | 48 | 12.6 | 35.4 | 11/1/2012 |
| Maniema | 45.3 | 9.2 | 36.1 | 9/1/2009 |
| Nord-Kivu | 38.8 | 2 | 36.8 | 4/1/2012 |
| Orientale | 36.3 | 1.1 | 35.2 | 9/1/2009 |
| Sud-Kivu | 58.1 | 3.4 | 54.7 | 4/1/2012 |

|  |  |  |  |  |  |  |
| --- | --- | --- | --- | --- | --- | --- |
|  |  |  |  |  |  |  |
