# Supplementary material for: The impact of an insecticide treated bednet campaign on all-cause child mortality: A geospatial impact evaluation from the Democratic Republic of Congo
Source: PLoS One. 2019 Feb 22;14(2):e0212890. doi: 10.1371/journal.pone.0212890 (PMC6386397; doi:10.1371/journal.pone.0212890)
Supplement: S5 Appendix — (DOCX) [file pone.0212890.s005.docx]

| **S5 Appendix: Analysis**  *Correlation of ITN usage increase with other health system indicators*  Finally, while our analysis uses subnational data on the timing of LLITN distribution, it is important to consider that our estimates could be confounded if the NMCP scaled up LLITN distribution and other interventions to the same provinces in the same months. We control for health aid investments at the province-month level, so such confounded interventions would need to be specific to domestic organizations in the DRC. Moreover, we can use indicators from the two available DHS rounds to test the correlation between increases in bednet usage and other health system indicators at the province level.  We construct the percentage point growth in children under 5 who slept under an ITN, children who took an ACT if they were feverish in the last two weeks, and children whose mothers sought advice or treatment for them if they were feverish.  The Spearman correlation between the ITN and ACT indicators is 0.37 (p=0.26), and between ITN coverage and seeking of advice or treatment is 0.26 (p=0.43).  These results suggest other interventions were not rolled out in the same geographic and temporal manner as the LLITN campaign, assuaging concerns that our estimates are confounded by these investments.  Nevertheless, we encourage the reader to interpret our estimates of the effect of the LLITN distribution on mortality as representing the effects of the overall program, including any activities (staff training or other investments) that went hand-in-hand with the net distribution. |  |  |  |  |  |  |
| --- | --- | --- | --- | --- | --- | --- |
|  |  |  |  |  |  |  |
